# An optimization scheduling model of multi-energy virtual power plants considering uncertainty constraints and multi-energy coupling characteristics

**DOI:** 10.1371/journal.pone.0343212

**Published:** 2026-03-03

**Authors:** Jia Lu, Junjie Wang, Jijun Liu, Youwu Liu

**Affiliations:** 1 Department of Electronic Engineering, Taiyuan Institute of Technology, Taiyuan, Shanxi, China; 2 Department of Production and Environmental Protection, China Huaneng Group CO., Ltd., Jinan, Shandong, China; 3 School of Economics and Management, Sanming University, Sanming, Fujian, China; University of Shanghai for Science and Technology, CHINA

## Abstract

Existing research on virtual power plants (VPPs) has not fully integrated the coupling relationships among electricity, heat, hydrogen, and carbon, and scheduling strategies under uncertainty conditions remain imperfect. To address this gap, this paper proposes an optimization scheduling model for a multi-energy virtual power plant (MEVPP) that incorporates uncertainty constraints and multi-energy coupling characteristics. The proposed model integrates biomass co-combustion carbon capture power plants (BCCPP), power-to-ammonia (P2A), and low-carbon chemical production (urea synthesis) within a unified stochastic VPP scheduling framework, achieving multi-energy synergy and flexible coupled operation involving electricity, heat, hydrogen, and carbon. A scenario generation method based on Latin hypercube sampling (LHS) is adopted to formulate a stochastic scheduling model aimed at maximizing the expected total system revenue under wind and solar uncertainties. Simulation results demonstrate that compared to the baseline scenario without carbon capture, the proposed model reduces CO₂ emissions by 38.5% (from 10,000 t to 6,150 t) and total costs by 75.1% (from $800,000 to $199,200) in the optimal scenario. Carbon trading price sensitivity analysis shows that emission reductions can reach 30–38% through constraint adjustments. These findings provide practical insights for system operators and policymakers in advancing low-carbon energy transitions, particularly for China’s dual-carbon goals.

## 1 Introduction

As the energy transition and the “dual carbon” goals advance, traditional power systems reliant on fossil fuels face challenges in achieving clean, low-carbon operations and efficient energy utilization [1]. Virtual Power Plants (VPPs) aggregate distributed energy resources to enhance system flexibility, serving as a core technology for this transition [2]. By integrating distributed generation, energy storage, and controllable loads, VPPs improve energy efficiency and enable participation in power and ancillary service markets, thereby enhancing peak-shaving and frequency regulation capabilities. Demonstration projects in Europe and the United States, such as the EU’s FENIX project and Denmark’s EDISON project, have advanced renewable integration [3,4]. In contrast, VPP research in China is relatively recent, with limited demonstration projects and a need for better alignment with local energy structures and market mechanisms. Recent studies extend VPPs to multi-energy coordination; for example, Yin et al. [5] proposed a multi-timescale model for power-gas VPPs with power-to-gas and demand response, while Xinfa et al. [6] optimized supply-demand balance in China’s power market.

Renewable sources like wind and solar offer clean energy but exhibit significant output fluctuations, impacting grid stability and leading to curtailment [7,8]. Multi-energy integrated systems coupling electricity, heat, gas, and hydrogen have emerged to improve efficiency and reduce emissions [9]. However, complex couplings and uncertainties in renewables and loads challenge VPP scheduling. Existing research employs robust and stochastic optimization or deep learning for forecasting, but limitations persist: insufficient consideration of electricity-heat-hydrogen-carbon couplings limits low-carbon benefits, and uncertainty strategies often lack robustness for economic-flexibility balance. While some studies integrate P2G and carbon capture, P2A and urea synthesis in stochastic VPP frameworks remain underexplored. The integration of P2A with carbon capture and multi-energy coupling in VPPs offers opportunities but requires models capturing electricity-carbon-hydrogen-chemical synergies, especially CO₂ utilization in chemicals. Robust frameworks are needed to balance economics and low-carbon goals under renewable stochasticity.

Unlike prior studies on MEVPPs that focus primarily on electricity–gas–heat couplings without explicit carbon recycling or chemical value addition, this study integrates the BCCPP as a core hub for flexible carbon flows, enabling a closed-loop electricity-carbon-hydrogen-chemical coupling mechanism with cascade heat utilization from electrolysis and synthesis processes to minimize waste and enhance efficiency. In contrast to existing P2G/P2A models that often treat CCS as an energy-intensive add-on without stochastic handling of renewables, our optimization formulation employs Latin Hypercube Sampling (LHS)-based scenario generation in a Mixed-Integer Linear Programming (MILP) framework to maximize expected revenue under correlated wind-PV uncertainties, achieving superior carbon reduction through integrated urea synthesis that repurposes captured CO₂ into marketable products, a feature underexplored in CCS-integrated scheduling.

This study proposes an optimized MEVPP scheduling model considering uncertainty and multi-energy coupling. Objectives include: (1) developing a framework integrating BCCPP, P2A, and chemical processes in a VPP; (2) formulating a stochastic model for wind-solar uncertainties; and (3) evaluating economic-environmental performance via case studies. The model constructs a framework with BCCPP, CHP, renewables, P2A, and chemical production, using flexible strategies like carbon capture and hydrogen blending. LHS-based scenarios enable stochastic optimization for economic-low-carbon balance.

This work contributes by: (1) proposing novel BCCPP-P2A-chemical integration in a unified VPP framework; (2) developing a stochastic model handling renewables while optimizing couplings; and (3) demonstrating significant emission reductions and cost savings compared to conventional systems. It provides insights for low-carbon scheduling and supports China’s energy transition goals.

## 2 Related work

### 2.1 VPPs research status

The VPP concept originated from the “virtual public utility” idea proposed by foreign scholars, positioning VPPs as independent market entities for aggregating distributed resources and scheduling flexibility [10]. Compared to conventional plants, VPPs offer greater clean energy integration, supporting low-carbon economies. As market participants, they provide ancillary services like peak shaving and frequency regulation. By integrating diverse units, VPPs optimize flexible resources for complementary advantages, enhancing system efficiency and low-carbon goals. VPPs are evolving from single-electricity systems to coordinated electricity-heat-gas multi-energy operations [5], spanning generation, transmission, conversion, and storage for collaborative optimization [11].

#### 2.1.1 Historical and International Developments in VPPs.

Since the early 21st century, Europe and North America have researched VPPs to manage distributed generation impacts on grids [12]. Research varies by region: Europe emphasizes integrating generation and storage for efficient supply, as in the EU’s FENIX project exploring grid technologies and market participation [3]. The U.S. prioritizes demand response (DR) for balancing renewables and achieving real-time supply-demand equilibrium [13]. With EV growth, Denmark’s 2009 EDISON project incorporated EVs as resources to optimize charging [4]. These regions are advanced, while China started later with fewer state-led projects due to sector constraints.

These international projects highlight VPP evolution from grid integration tools to flexible market participants, but often overlook multi-energy couplings, a gap addressed in recent studies like ours through stochastic modeling under uncertainty.

#### 2.1.2 Recent optimization methods for VPPs.

Extensive VPP research integrates units like thermal, wind, PV, storage, and turbines [14], improving economic performance in energy and reserve markets while enhancing flexibility. Zhao et al. [15] proposed model predictive control with LSTM for short-term load/renewable forecasting, boosting utilization and reducing error impacts. Luo [16] used multi-stage robust optimization for uncertainty management and bidding strategies. Wen et al. [17] aggregated renewables in Northwest China for multi-timescale robust stochastic scheduling, enhancing benefits and consumption.

These methods underscore shifts toward robust and predictive uncertainty handling in VPPs; however, they focus on electricity-only systems, limiting multi-energy applicability, which our model extends by incorporating hydrogen and carbon couplings for enhanced low-carbon performance.

### 2.2 Current status of research on multi-energy coupled VPPs

Amid energy shortages and pollution, transformations balance efficiency and low emissions [18]. Renewables like wind/solar are central but unstable, causing curtailment [19]. Multi-energy systems integrate electricity, heat, gas, and clean energy for complementarity via grids and networks [20,21].

#### 2.2.1 Uncertainty in VPP scheduling.

Rong & Kuang [22] used CVaR and NSGA-III for wind randomness. Liu et al. [23] proposed two-layer robust models for multi-energy stability. Gao et al. [24] applied PDF-based scenarios for reliability. Shi et al. [25] integrated robust theory with demand response. Xiao et al. [26] considered correlations and emissions limits. Zhou & Li [27] used two-stage robust with adjustable parameters. Son et al. [28] employed Monte Carlo and aggregation. Collectively, these studies [22–28] illustrate probabilistic, robust, and scenario-based techniques for managing uncertainties, which enhance system reliability but can introduce computational complexity or conservatism; our LHS-based stochastic model builds on these by providing a balanced approach that maximizes revenue while accommodating multi-energy synergies.

#### 2.2.2 Multi-energy coupling in VPPs.

Multi-energy VPPs optimize couplings for flexibility. Wan et al. [29] modeled electricity-gas-heat-carbon synergies under markets. Zhang et al. [30] designed multi-source heating with transients. These advance coordination but underexplore carbon-hydrogen-chemical integrations.

#### 2.2.3 Carbon capture in multi-energy systems.

Li et al. [31] modeled microgrids with carbon capture, CCHP, and P2G. Cho et al. [32] optimized VPP bidding with Power-to-X. Liu et al. [33] used game theory for P2G-CCS synergies.

These integrations [31–33] highlight the potential of carbon capture for low-carbon VPPs, yet they often treat CCS as an add-on rather than a core coupled element, focusing on electricity-gas-heat synergies without explicit chemical value chains or stochastic uncertainty management; our model advances this by embedding BCCPP as a flexible, biomass-enhanced carbon source within a unified stochastic MILP framework, positioning P2A and urea synthesis as integral mechanisms for CO₂ recycling into high-value products, unlike the deterministic or robust approaches in [31–33] that lack LHS-based correlated scenario handling for wind-PV uncertainties, thereby enabling more robust multi-energy coupling and superior economic-low-carbon trade-offs.

## 3 Methodology

### 3.1 MEVPP framework considering multi-energy coupling characteristics and low-carbon chemical production

**Fig 1:**
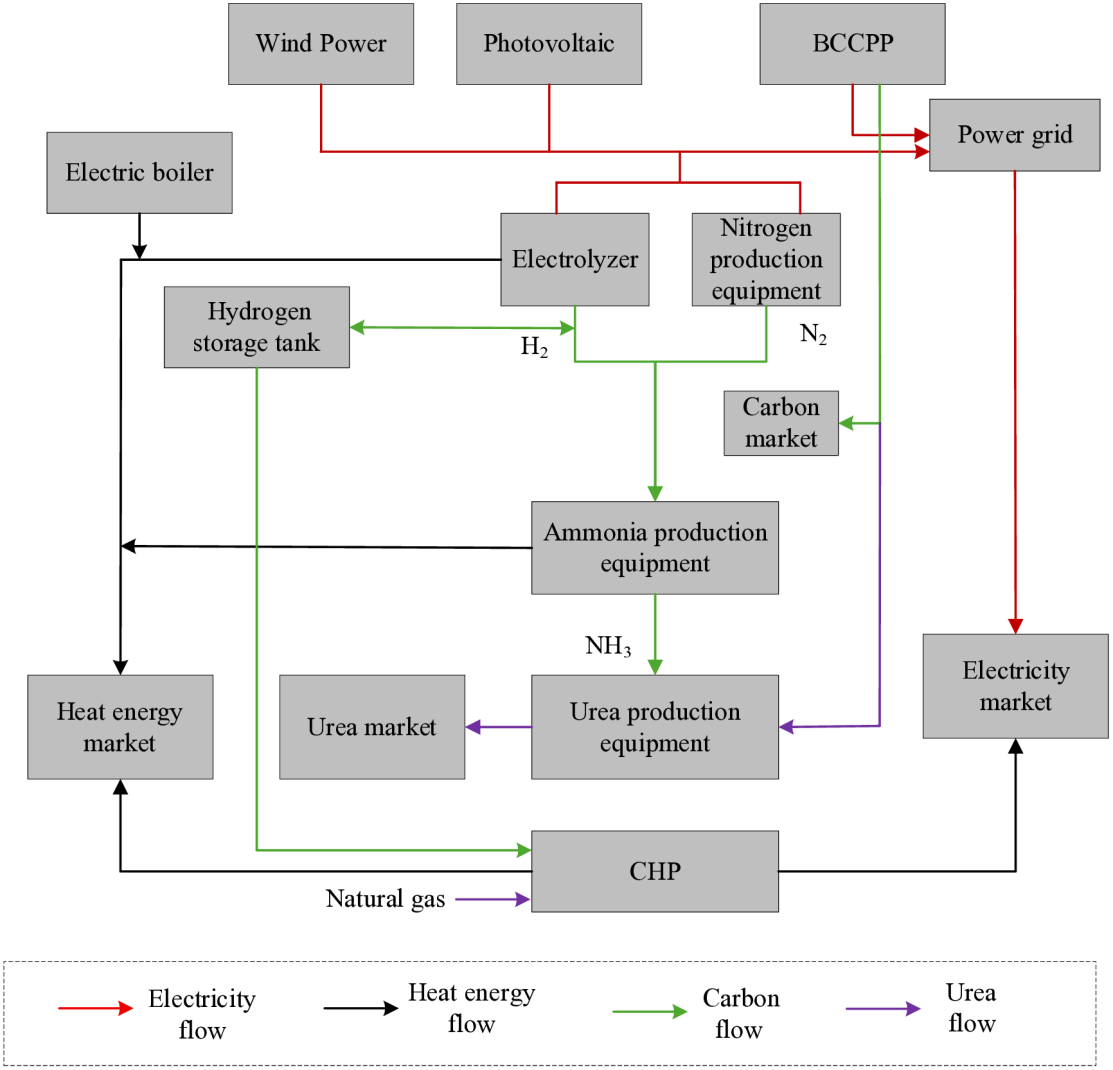
The structure of the multi-energy virtual power plant (MEVPP). The figure illustrates the integrated electricity–heat–hydrogen–carbon system, including wind power, PV generation, CHP, BCCPP, P2A, and chemical production units. Arrows represent energy and material flows among electricity, heat, hydrogen, and CO₂ subsystems, highlighting the coupling relationships and closed-loop operation enabled by carbon capture and utilization.

The MEVPP framework proposed in this study integrates multiple low-carbon technologies to address the challenges of high renewable penetration and carbon reduction in energy systems. As illustrated in Fig 1, the MEVPP incorporates electricity, heat, hydrogen, and carbon coupling relationships. Electricity demand is supplied by wind power, photovoltaic (PV) power, CHP, and BCCPP. Heat demand is met through chemical production, CHP, and electric boilers. Surplus wind and PV resources are channeled into electric boilers and chemical processes for efficient utilization. Captured CO₂ from carbon capture is partly used in urea production for revenue generation, with the excess sold in the carbon market. Specifically, the framework highlights bidirectional interactions: for instance, excess electricity from renewables flows to the P2A unit for hydrogen production, which in turn interacts with captured CO₂ from BCCPP to enable chemical synthesis (e.g., ammonia and urea), while waste heat from these processes recirculates to support heat demands via CHP or boilers. This creates a closed-loop energy flow that reduces waste, with carbon serving as a linking medium between power generation and chemical subsystems, promoting overall system resilience and low-carbon efficiency.

This framework promotes synergistic operation among diverse energy units, enhancing system flexibility and low-carbon performance while mitigating renewable curtailment. By leveraging “coal-fired+” strategies, the MEVPP transforms traditional high-emission units into efficient, multi-energy hubs that support both economic viability and environmental goals.

#### 3.1.1 Flexible operation model of BCCPP.

**Fig 2:**
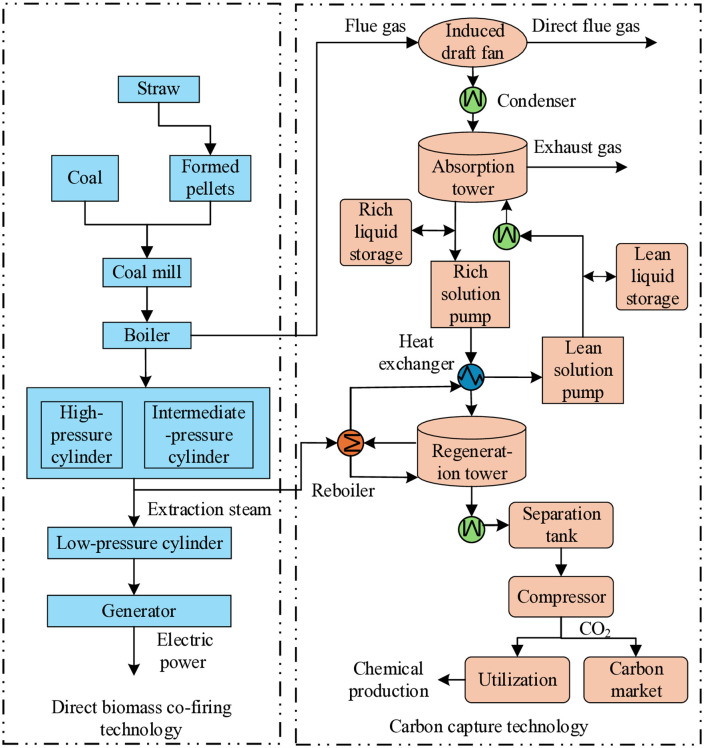
System structure of the BCCPP. Coal and biomass are co-fired to generate gross electrical power, part of which is consumed by the carbon capture unit, resulting in net power output. The figure shows fuel input streams, flue gas routing to the capture subsystem, and captured CO₂ flows directed to storage or utilization, illustrating the interaction between power generation and carbon capture processes.

Traditional coal-fired units pose environmental challenges due to high emissions, hindering low-carbon energy system operations. Biomass, with its renewable and low-carbon properties, enables cleaner fuel substitution through co-combustion with coal, reducing overall emissions and supporting sustainable development. Direct biomass blending stands out for its low retrofit costs, minimal disruption to existing units, high efficiency, and broad applicability. In this study, biomass direct coupling is combined with carbon capture to upgrade coal-fired units into BCCPP, achieving dual benefits of fuel substitution and carbon removal. The BCCPP structure, as shown in Fig 2, features biomass and coal inputs, with outputs comprising net power and carbon capture energy consumption. The diagram emphasizes key interactions, such as the co-combustion process where coal and biomass fuels mix to generate gross power, followed by the carbon capture subsystem that deducts energy for CO₂ removal, resulting in net power output to the grid. Flows include fuel input streams converging in the boiler, flue gas directed to the capture unit, and captured CO₂ routed for storage or utilization in downstream chemical processes, allowing flexible adjustments based on emission constraints and grid demands. This integration configuration allows BCCPP to provide flexible regulation, adjusting output based on grid demands and emission constraints, thereby contributing to system stability.

The simplified BCCPP energy balance model incorporates both the fuel co-combustion process and the carbon capture dynamics. The total gross power output from the BCCPP unit is derived from the weighted contributions of coal and biomass fuels, accounting for their respective heating values and combustion efficiencies. Biomass is blended at a variable ratio $\alpha_{t}$ to optimize low-carbon operation. The gross power $P_{\text{BCCPP},t}^{\text{gross }}$ is then reduced by the energy penalty associated with carbon capture $E_{\text{capture},\;t}$, yielding the net power output $P_{\text{BCCPP},t}^{\text{net }}$:


$$\begin{array}{c} P_{\text{BCCPP},t}^{\text{gross}}=\eta_{\text{coal }}\cdot Q_{\text{coal },t}\cdot \left(\text{1}-\alpha_{t}\right)+\eta_{\text{bio }}\cdot Q_{\text{bio },t}\cdot \alpha_{t}\\ P_{\text{BCCPP},t}^{\text{net }}=P_{\text{BCCPP},t}^{\text{gross}}-E_{\text{capture },t} \end{array}$$ (1)

Where $\alpha_{t}$ is the biomass blending ratio at time *t* ($\text{0}\leq \alpha_{t}\leq \text{1}$), and $Q_{\text{coal},\;t},Q_{\text{bio},\;t}$ are the heating values of coal and biomass inputs, respec*t*ively.

Carbon capture is modeled flexibly for emission optimization, where the amount of CO₂ captured $M_{\text{CO}\text{2},\text{ capture },t}$ is proportional to the total CO₂ emissions from combustion, adjusted by the capture efficiency $\eta_{\text{capture }}$ and a split ratio $\beta_{t}$ for utilization (e.g., in chemical processes) versus storage. The energy consumption for capture $E_{\text{capture },t}$ is a function of the captured amount and specific energy intensity $\gamma_{\text{capture }}$:


$$\begin{array}{c} M_{\text{CO}\text{2},\text{ capture },t}=\eta_{\text{capture }}\cdot \beta_{t}\cdot \left(I_{\text{coal }}\cdot Q_{\text{coal },t}\cdot \left(\text{1}-\alpha_{t}\right)+I_{\text{bio }}\cdot Q_{\text{bio },t}\cdot \alpha_{t}\right)\\ E_{\text{capture },t}=\gamma_{\text{capture }}\cdot M_{\text{CO}\text{2},\text{ capture },t} \end{array}$$ (2)

With energy use derived from the capture process, where excess captured CO₂ $\begin{array}{c} (M_{\text{CO}\text{2},\text{ excess},\;t}=M_{\text{CO}\text{2},\text{ capture},\;t}-M_{\text{CO}\text{2},\text{ stored},\;t}-M_{\text{CO}\text{2},\text{ utilized},\;t})\; \end{array}$ can be sold in the carbon market.

These models ensure BCCPP contributes to upward and downward flexibility, with carbon capture adjusting based on grid needs and emission targets, simplifying analysis while capturing essential low-carbon mechanics.

In Equations (1)–(2), $P_{\text{BCCPP},t}^{\text{gross}}$ and $P_{\text{BCCPP},t}^{\text{net }}$ denote BCCPP total gross power and net output at time *t*, respectively; $E_{\text{capture },t}$ denotes carbon capture energy consumption at time *t*; $\eta_{\text{coal}}$ and $\eta_{\text{bio}}$ are combus*t*ion efficiencies for coal and biomass (typical values: $\eta_{\text{coal}}$ =0.40, $\eta_{\text{bio}}$ =0.35, based on typical pulverized coal and biomass co-combustion systems); $Q_{\text{coal},t}$ and $Q_{\text{bio},t}$ are heating values of coal and biomass inputs at time *t*; $M_{\text{CO}\text{2},\text{ capture },t}$, $M_{\text{CO}\text{2},\text{ stored },t}$, and $M_{\text{CO}\text{2},\text{ utilized },t}$ are captured, s*t*ored, and utilized CO₂ amounts at time *t*, respectively; $\eta_{\text{capture }}$ is carbon capture efficiency (typical value: 0.90, consistent with post-combustion capture systems); $\gamma_{\text{capture}}$ is energy consumption intensity for carbon capture (typical value: 0.4 MWh/t CO₂, within the range of 0.3–0.5 MWh/t CO₂ for amine-based capture systems); $\beta_{t}$ is the split ratio for CO₂ utilization versus storage at *t*ime t (0≤$\beta_{t}$≤1); $I_{\text{coal }}$ and $I_{\text{bio }}$ are CO₂ emission intensities for coal and biomass (typical values: $I_{\text{coal }}$ =0.35 t CO₂/MWh, $I_{\text{bio }}$ =0.05 t CO₂/MWh, where biomass emissions represent direct combustion emissions only, assuming carbon-neutral biomass lifecycle; when carbon capture is applied to biomass co-combustion, the captured CO₂ from biomass combustion is treated as nega*t*ive emissions, enabling net carbon removal when combined with storage or utilization); $P_{\text{BCCPP },\text{ max }}$ is the maximum power output of BCCPP. The parameter values are based on literature-reported ranges for commercial systems: combustion efficiencies align with pulverized coal boilers ($\eta_{\text{coal}}$ = 0.35–0.42) and biomass co-firing systems ($\eta_{\text{bio}}$ = 0.30–0.38); carbon capture efficiency (0.85–0.95) and energy intensity (0.3–0.5 MWh/t CO₂) reflect post-combustion amine-based capture performance; emission intensities follow IPCC guidelines for fossil fuels ($I_{\text{coal}}$ = 0.34–0.36 t CO₂/MWh) and biomass direct combustion ($I_{\text{bio}}$ = 0.03–0.06 t CO₂/MWh, direct emissions only under carbon-neutral lifecycle assumption).

#### 3.1.2 Electricity-carbon-hydrogen-chemical coupling relationships and detailed modeling.

**Fig 3:**
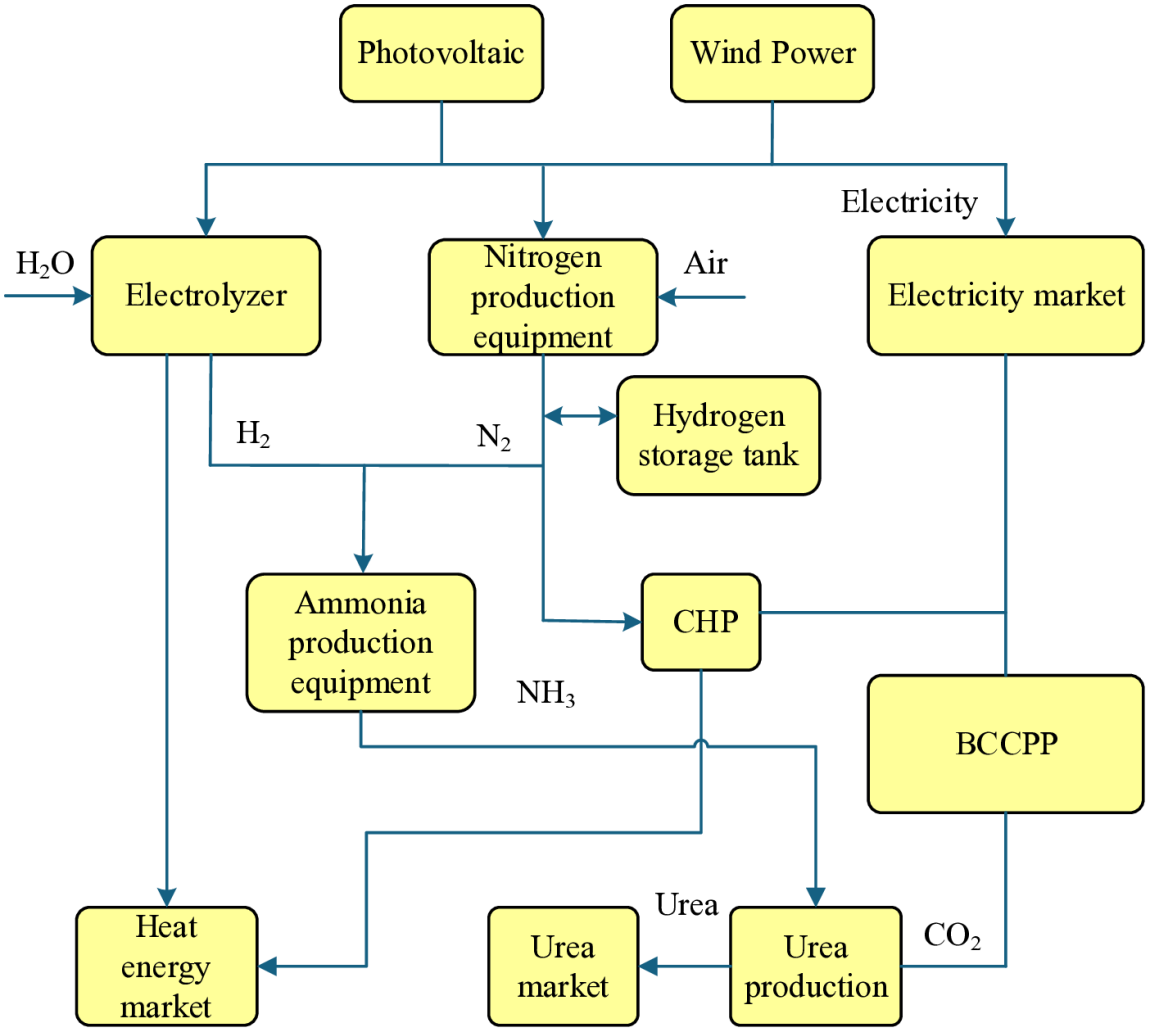
Energy flow relationships in the electricity–carbon–hydrogen–chemical coupling system. Surplus electricity is converted into hydrogen via electrolysis, captured CO₂ is combined with hydrogen for ammonia and urea synthesis, and exothermic heat from chemical reactions is recovered and reused by CHP units or electric boilers. The figure highlights cascade heat utilization and multi-energy coupling among electricity, carbon, hydrogen, and chemical subsystems.

High renewable integration introduces volatility, which P2A addresses by converting surplus electricity into ammonia, enabling low-carbon chemical production and aiding the chemical sector’s transition. The MEVPP’s multi-energy coupling system, depicted in Fig 3, facilitates heat energy flows across electricity, carbon, hydrogen, and chemicals, optimizing cascade utilization for improved efficiency and reduced waste. In particular, the figure illustrates dynamic interactions: electricity from renewables or BCCPP powers electrolysis for hydrogen production, which combines with nitrogen and captured CO₂ in the synthesis loop to form ammonia and urea; exothermic heat from synthesis flows back to support CHP or boiler operations, creating a cascade where waste energy from one subsystem (e.g., electrolysis heat) enhances another (e.g., chemical heating), thereby reducing external energy inputs and fostering a synergistic, low-carbon cycle.

This coupling allows captured CO₂ and green hydrogen to feed into chemical processes, creating value-added products such as urea while reducing reliance on external carbon and hydrogen sources. The design emphasizes energy ladder usage, where waste heat from one process supports another, maximizing overall utilization and aligning with low-carbon objectives.

The electrolyzer model for hydrogen production starts from the electrical input $P_{\text{elec},\text{in},\;t},$ converted to hydrogen output $M_{\text{H}\text{2},\text{ out },t}$ via electrolysis efficiency $\eta_{\text{electrolyzer }}$, with associated heat generation $Q_{\text{heat},\text{elect},\;t}$ recovered for downstream use. Hydrogen storage buffers production variability:


$$\begin{array}{c} M_{\text{H}\text{2},\text{ out },t}=\eta_{\text{electrolyzer }}\cdot \frac{P_{\text{elec},\text{in },t}\cdot \Delta t}{HHV_{\text{H}\text{2}}}\mathrm{\backslash vspace}1mm\\ Q_{\text{heat},\text{elect },t}=\left(\text{1}-\eta_{\text{electrolyzer }}\right)\cdot P_{\text{elec},\text{in },t}\cdot \Delta t\mathrm{\backslash vspace}1mm\\ M_{\text{H}\text{2},\text{ stored },t}=M_{\text{H}\text{2},\text{ stored },t-\text{1}}+\eta_{\text{storage }}\cdot M_{\text{H}\text{2},\text{ out },t}-\frac{M_{\text{H}\text{2},\text{ out },t}}{\eta_{\text{storage }}}\cdot \left(\text{1}-SOC_{\text{limit }}\right) \end{array}$$ (3)

Where $SOC_{\text{limit }}$ enforces storage bounds ($\text{0}\leq SOC_{t}\leq \text{1}$). Pressure swing adsorption (PSA) produces nitrogen from air, serving as a key enabler for downstream synthesis without detailed standalone modeling here.

Ammonia synthesis via the Haber-Bosch process consumes hydrogen $M_{\text{H}\text{2},\text{in},t}$ and nitrogen, incorporating captured CO₂ for urea production. The ammonia output $M_{\text{NH}\text{3},\text{ out },t}$ is efficiency-limited, generating heat $Q_{\text{heat},\text{ammonia},\;t}$, which cascades to CHP or boilers. Urea synthesis follows:


$$\begin{array}{c} M_{\text{NH}\text{3},\text{ out },t}=\eta_{\text{ammonia }}\cdot min\left(\frac{M_{\text{H}\text{2},\text{ in },t}}{\text{3}},M_{\text{N}\text{2},\text{ in },t}\right)\\ M_{\text{urea},\text{out },t}=\eta_{\text{urea }}\cdot min\left(M_{\text{NH}\text{3},\text{ out },t},\frac{M_{\text{CO}\text{2},\text{ utilized },t}}{\text{1}}\right)\cdot \mu_{\text{urea }}\\ Q_{\text{heat},\text{ammonia },t}=\Delta H_{\text{reaction }}\cdot M_{\text{NH}\text{3},\text{ out },t} \end{array}$$ (4)

Where $\Delta H_{\text{reaction }}$ is the exothermic heat of reaction, and $\mu_{\text{urea }}$ is the mass conversion factor.

CHP with hydrogen blending enhances flexibility by adjusting the fuel mix, producing electricity $P_{\text{CHP},\text{elec},\text{t }}$ and heat $Q_{\text{CHP},\text{ heat },t}$:


$$\begin{array}{c} P_{\text{CHP},\text{ elec },t}=\eta_{\text{CHP},\text{ elec }}\cdot \left(Q_{\text{gas},t}+HHV_{\text{H}\text{2}}\cdot M_{\text{H}\text{2},\text{ blended },t}\right)\\ Q_{\text{CHP},\text{ heat },t}=\eta_{\text{CHP},\text{ heat }}\cdot \left(\text{1}-\eta_{\text{CHP},\text{ elec }}\right)\cdot \left(Q_{\text{gas },t}+HHV_{\text{H}\text{2}}\cdot M_{\text{H}\text{2},\text{ blended },t}\right)\\ M_{\text{H}\text{2},\text{ blended },t}=\delta_{t}\cdot \frac{Q_{\text{gas},t}}{HHV_{\text{H}\text{2}}} \end{array}$$ (5)

These consolidated models capture essential coupling dynamics, focusing on energy conversion and storage to facilitate reader understanding while supporting low-carbon chemical pathways. The derivations assume steady-state operation and ideal gas laws for simplicity; heat recovery efficiencies are parameterized to reflect cascade utilization.

In Equations (3)–(5), $M_{\text{H}\text{2},\text{ out },t}$ denotes electrolyzer hydrogen output at time *t*; $P_{\text{elec},\text{in},\;t}$ denotes electrical input *t*o electrolyzer at time *t*; $Q_{\text{heat},\text{ elect },t}$ denotes heat generation from electrolysis at time *t*; $\eta_{\text{electrolyzer}}$ is elec*t*rolyzer efficiency (typical value: 0.70, representative of alkaline electrolyzers); $\eta_{\text{storage }}$ is hydrogen storage efficiency (typical value: 0.95, for compressed hydrogen storage systems); $V_{\text{H}\text{2},\text{ storage }}$ is hydrogen storage capacity; $M_{\text{NH}\text{3},\text{ out },t}$ deno*t*es ammonia output at time *t*; $M_{\text{H}\text{2},\text{in},t}$ denotes hydrogen input to ammonia synthesis at time *t*; $Q_{\text{heat },\text{ ammonia },t}$ denotes heat generation from ammonia synthesis at time *t*; $\eta_{\text{ammonia }}$ is ammonia synthesis efficiency (typical value: 0.85, for Haber-Bosch process); $\mu_{\text{urea }}$ is urea synthesis efficiency (typical value: 0.90, for industrial urea production); $\mu_{\text{urea }}$ is mass conversion factor for urea (typical value: 1.47, based on stoichiometric ratios); $\Delta H_{\text{reaction }}$ is exothermic heat of reac*t*ion for ammonia synthesis (typical value: −46.1 MJ/kg NH₃, standard thermodynamic value); $P_{\text{CHP},\text{ elec },t}$ denotes CHP electrical output at time *t*; $Q_{\text{CHP},\text{ heat },t\;}$ denotes CHP heat output at time *t*; $\eta_{\text{CHP},\text{ elec }}$ is CHP electrical efficiency (typical value: 0.40); $\eta_{\text{CHP},\text{ heat }}$ is CHP heat efficiency (*t*ypical value: 0.45); $HHV_{\text{H2}}\;$ denotes higher heating value of hydrogen (typical value: 141.8 MJ/kg, standard thermodynamic value); $\delta_{t}$ denotes hydrogen blending ratio at time t (0 ≤ δ_t ≤ 0.3, limited by gas turbine combustion stability); $SOC_{\text{t}}$ denotes state of charge at time t, with bounds 0 ≤ SOC_“t” ≤ 1. The electrolyzer efficiency (0.65–0.75 for alkaline systems), ammonia synthesis efficiency (0.80–0.90 for Haber-Bosch process), and hydrogen storage efficiency (0.90–0.98 for compressed systems) are consistent with industrial practice and technical literature on power-to-gas and chemical synthesis applications.

### 3.2 MEVPP stochastic low-carbon scheduling model based on wind and solar scenario generation

Wind and PV uncertainties in scheduling necessitate robust handling to maintain system reliability. This study uses scenario analysis to model these uncertainties, constructing a stochastic model that maximizes revenue under energy balance, coupling, and operational constraints, thereby balancing economic and environmental goals.

#### 3.2.1 Scenario generation for wind and solar uncertainties.

Prediction errors for wind and PV follow normal distributions with zero mean and standard deviations of 10% and 8% for wind and PV, respectively. The standard deviation values are consistent with forecasting error ranges observed in operational practice and reported in renewable energy forecasting studies, where wind power forecasting errors typically range from 8–12% and PV forecasting errors from 6–10% for day-ahead horizons. To capture the correlation between wind and PV outputs, a Gaussian copula function is employed with a correlation coefficient of 0.3, reflecting the weak complementary relationship between wind and solar resources documented in meteorological studies, where diurnal and seasonal patterns lead to mild negative or low positive correlations. LHS generates diverse scenarios for better coverage, initially producing 1000 scenarios to ensure sufficient coverage of the uncertainty space, following established practices in stochastic optimization for power systems. Subsequently, synchronous backward reduction (SBR) is applied to reduce the scenario set to 4 representative scenarios while preserving the statistical properties of the original distribution. The selection of 4 scenarios is based on a trade-off between computational efficiency and solution quality: empirical tests indicate that increasing scenarios beyond 4 yields diminishing improvements in solution accuracy (less than 2% difference in expected revenue) while significantly increasing computation time, consistent with scenario reduction convergence behavior observed in multi-stage stochastic optimization literature. The four scenarios represent typical operational conditions: high wind-high PV, high wind-low PV, low wind-high PV, and low wind-low PV, with probabilities of 0.30, 0.25, 0.25, and 0.20, respectively, derived from the scenario reduction process that preserves the first two moments of the original distribution.


$$P_{\text{renew },t}^{p}=P_{\text{renew },\text{ pred },t}\cdot \left(\text{1}+\epsilon_{t}^{p}\right)$$ (6)

Where $\epsilon_{t}^{p}$ is the error term for scenario *p* at time *t*, sampled to reflect correlations be*t*ween wind and PV (e.g., via copula functions for joint distribution). $P_{\text{renew },t}^{p}$ is renewable power (wind + PV) in scenario *p* at time *t*; $P_{\text{renew},\text{ pred},\;t}$ is the deterministic prediction; $\epsilon_{t}^{p}$ is the normalized error.

#### 3.2.2 MEVPP stochastic scheduling model.

***Objective function.*** The model maximizes expected revenue by aggregating benefits and costs over scenarios and time periods:


$$max\sum_{p=\text{1}}^{S}\;\pi_{p}\sum_{t=\text{1}}^{T}\;C_{t}^{p}$$ (7)

Where $C_{t}^{p}$ is the net revenue at time *t* in scenario *p*.

Energy/chemical/carbon sales consolidate as:


$$R_{\text{sales },t}^{p}=\lambda_{\text{elec }}\cdot P_{\text{sold},\text{elec},\;t}^{p}+\lambda_{\text{heat }}\cdot Q_{\text{sold},\text{heat },t}^{p}+\lambda_{\text{urea }}\cdot M_{\text{urea},\text{out },t}^{p}+r\cdot M_{\text{CO2},\text{excels},t\;}^{p}$$ (8)

$$R_{\text{chem},t}^{p}=\lambda_{\text{H}\text{2}}\cdot M_{\text{H}\text{2},\text{sold},t}^{p}$$ (9)

Carbon trading nets quotas and emissions:


$$C_{\text{carbon },t}^{p}=r\cdot \left(Q_{\text{CO}\text{2},\text{ quota },t}-E_{\text{CO}\text{2},\text{ actual },t}^{p}\right)$$ (10)

Where positive values indicate revenue from surplus quotas.

Operation costs aggregate:


$$\begin{array}{c} K_{\text{op},t}^{p}=c_{\text{fuel}}\cdot \left(Q_{\text{coal},t}^{p}+Q_{\text{bio},t}^{p}+Q_{\text{gas},t}^{p}\right)+c_{\text{maint}}\cdot \sum {\mathrm{\backslash nolimits}}_{i}\;I_{i}\cdot P_{i,t}^{p} \end{array}$$ (11)

Curtailment and depreciation:


$$\begin{array}{c} K_{\text{curt},\text{dep},t}^{p}=\kappa_{\text{curt}}\cdot \left(P_{\text{wind},\text{curt},t}^{p}+P_{\text{PV},\text{curt},t}^{p}\right)+d\cdot \sum {\mathrm{\backslash nolimits}}_{i}\;\frac{P_{i,max}\cdot T}{I_{i}} \end{array}$$ (12)

These streamlined objectives focus on net revenue maximization, reducing redundancy by grouping similar terms. The stochastic expectation accounts for uncertainty, with $\pi_{p}$ weighting scenarios.

In Equations (7)–(12), S denotes the number of scenarios; T denotes the number of time periods; $C_{t}^{p}$ denotes net revenue at time t in scenario p; $\pi_{p}$denotes the probability of scenario p; $R_{\text{sales },t}^{p}$ denotes total sales revenue at time t in scenario p; $K_{\text{op},t}^{p}$ denotes operation cost at time t in scenario p; $\lambda_{\text{elec}}$ denotes electricity price (typical value: 50 $/MWh); $\lambda_{\text{heat}}$ denotes heat price (typical value: 30 $/MWh); $\lambda_{\text{urea }}$ denotes urea price (typical value: 300 $/t); λ_H2 denotes hydrogen price (typical value: 3 $/kg); r denotes carbon trading price (typical value: 19.8 $/t CO₂); $P_{\text{sold},\text{elec },t}^{p}$ denotes sold electricity quantity at time t in scenario p; $Q_{\text{sold},\text{heat },t}^{p}$ denotes sold heat quantity at time t in scenario p; $M_{\text{urea},\text{out },t}^{p}$ denotes urea output at time t in scenario p; $M_{\text{H}\text{2},\text{sold},t}^{p}$ denotes sold hydrogen quantity at time t in scenario p; $M_{\text{CO2},\text{excels},t\;}^{p}$ denotes excess CO₂ sold in carbon market at time t in scenario p; $E_{\text{CO}\text{2},\text{ actual},\;t}^{p}$ denotes actual CO₂ emissions at time t in scenario p; $Q_{\text{CO}\text{2},\text{ quota},\;t}$ denotes CO₂ emission quota at time t; $C_{\text{fuel }}$ denotes fuel cost coefficient (typical values: 20 $/MWh for coal, 25 $/MWh for biomass, 30 $/MWh for natural gas); $C_{\text{maint }}$denotes maintenance cost coefficient (typical value: 5 $/MWh); $\kappa_{\text{curt }}$ denotes wind curtailment penalty coefficient (typical value: 40 $/(MW•h)); d denotes depreciation cost coefficient; $I_{i}$ denotes equipment count for unit i; $P_{i,max}$ denotes maximum power output of unit i; $P_{\text{wind},\text{curt},t}^{p}$ and $P_{\text{PV},\text{curt},t}^{p}$ denote curtailed wind and PV power at time t in scenario p, respectively.

***Constraints.*** Power balance constraints ensure equilibrium across energy forms (electricity, heat, hydrogen) for each scenario and time:


$$\begin{array}{cc} & P_{\text{load},\text{elec },t}^{p}=P_{\text{wind },t}^{p}+P_{\text{PV},t}^{p}+P_{\text{BCCPP},t}^{p}+P_{\text{CHP},\text{ elec },t}^{p}+P_{\text{boiler},\text{ elec },t}^{p}\\ & -P_{\text{P}\text{2}\text{A},\text{ elec },t}^{p}-P_{\text{capture},\text{elec },t}^{p}\\ & Q_{\text{load},\text{ heat },t}^{p}=Q_{\text{chem},\text{ heat },t}^{p}+Q_{\text{CHP},\text{ heat },t}^{p}+Q_{\text{boiler},\text{ heat },t}^{p}-Q_{\text{recovery},\text{ heat },t}^{p}\\ & M_{{\text{H}}_{\text{2}},\text{ produced },t}^{p}+M_{{\text{H}}_{\text{2}},\text{ stored },t-\text{1}}^{p}=M_{\text{load},\;,{\text{H}}_{\text{2}},t}^{p}+M_{{\text{H}}_{\text{2}},\text{ blended },t}^{p}+M_{{\text{H}}_{\text{2}},\text{ sold },t}^{p}+M_{{\text{H}}_{\text{2}},\text{ stored },t}^{p} \end{array}$$ (13)

Renewable and unit limits consolidate operational bounds:


$$\text{0}\leq P_{\text{renew },t}^{p}\leq P_{\text{renew },max,t}^{p},P_{i,min}\leq P_{i,t}^{p}\leq P_{i,max},\forall i\in \{\text{BCCPP},\text{CHP},\text{ boiler },\text{ capture}\}$$ (14)

Where $P_{\text{renew },t}^{p}$ denotes maximum available renewable power at time t in scenario p; $P_{i,min}$ and $P_{i,max}$ denote minimum and maximum power output limits for unit i, respectively.

Ramp rate constraints ensure that unit output changes respect physical limitations:


$$-DR_{i}\leq P_{(}i,t)p-P_{(}i,t-\text{1})p\leq UR_{i},\forall i\in \{BCCPP,CHP,boiler\}$$ (15)

Where $DR_{i}$ and $UR_{i}$ denote downward and upward ramp rates for unit i (typical values: DR_BCCPP = UR_BCCPP = 50 MW/h, DR_CHP = UR_CHP = 30 MW/h, DR_boiler = UR_boiler = 20 MW/h).

Storage constraints for hydrogen storage ensure operational bounds:


$$SOC_{min}\;\leq SOC_{t}\leq SOC_{max}\;,M_{H\text{2},stored,\text{0}}\;=M_{H\text{2},stored,T}$$ (16)

Where $SOC_{min}$ and $SOC_{max}$ are minimum and maximum state-of-charge limits (typical values: $SOC_{min}$=0.1, $SOC_{max}$=0.9), and the final condition ensures daily energy balance for hydrogen storage.

In Equations (13)–(16), $P_{\text{load},\text{elec },t}^{p}$, $Q_{\text{load},\text{ heat },t}^{p}$, and $M_{\text{load},\;{\text{H}}_{\text{2}},t}^{p}$ denote electricity, heat, and hydrogen demand at time *t* in scenario *p*, respectively; $P_{i,min}$ and $P_{i,max}$ denote minimum and maximum power output limits for unit *i*; $M_{{\text{H}}_{\text{2}},\text{ produced },t}^{p}$ denotes hydrogen production from electrolysis at time *t* in scenario *p*; $M_{{\text{H}}_{\text{2}},\text{ stored },t}^{p}$ denotes hydrogen stored at time *t* in scenario *p*; $M_{{\text{H}}_{\text{2}},\text{ blended},t}^{p}$ and $M_{{\text{H}}_{\text{2}},\text{ sold },t}^{p}$ denote hydrogen used for blending in CHP and sold externally at time *t* in scenario *p*, respectively. The hydrogen balance equation (third line of Equation 13) ensures that hydrogen production plus storage at the previous time step equals demand plus blending, sales, and storage a*t* the current time step. The model assumes steady-state operation for chemical processes and excludes s*t*art-up/shutdown costs for simplicity, focusing on operational flexibility and multi-energy coupling dynamics. All constraints are linear, ensuring MILP compatibility.

#### 3.2.3 Model solving process.

**Fig 4:**
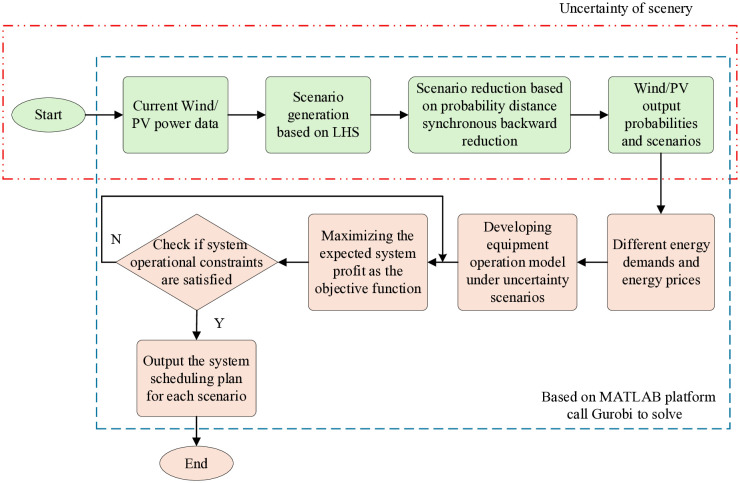
Flowchart of the MEVPP stochastic scheduling model solution. The procedure includes wind and PV scenario generation using LHS, scenario reduction, formulation of the MILP model, and solution using the YALMIP–Gurobi framework.

The MEVPP stochastic low-carbon scheduling model constructed in this paper is a MILP model. To enhance model solving speed and efficiency, this study employs the yalmip toolbox on the MATLAB platform to invoke Gurobi for solution. Gurobi’s high-efficiency solving capabilities and MATLAB’s modeling convenience provide distinct advantages in addressing this problem. All tests were conducted on a computer equipped with an Intel Core i7-11800H CPU and 16GB of RAM. The proposed model solution workflow is illustrated in Fig 4.

## 4 Result analysis and discussion

### 4.1 Basic data

All simulation results presented in this section are based on a 24-hour scheduling horizon, representing a typical daily operational cycle. The predicted values for electricity load, heat load, hydrogen demand, wind power, and PV power are shown in Table 1. The data in Table 1 represent a typical daily profile. Although synthetic due to the lack of publicly available detailed data, they are based on realistic operational patterns from similar multi-energy systems. The penalty cost coefficient for wind curtailment is set to 40 $/(MW•h), the carbon trading cost coefficient is 19.8 $/t, and the CO₂ storage cost coefficient is 5 $/t. Key economic and technical parameters include: electricity price 50 $/MWh, heat price 30 $/MWh, urea price 300 $/t, hydrogen price 3 $/kg, coal price 20 $/MWh, biomass price 25 $/MWh, natural gas price 30 $/MWh, electrolyzer efficiency 0.70, ammonia synthesis efficiency 0.85, urea synthesis efficiency 0.90, carbon capture efficiency 0.90, and carbon capture energy intensity 0.4 MWh/t CO₂. To account for the uncertainties in wind and PV outputs, 1000 initial scenarios are generated using LHS, and then reduced to 4 representative scenarios with associated probabilities via synchronous backward reduction (probabilities: 0.30, 0.25, 0.25, and 0.20 for high wind-high PV, high wind-low PV, low wind-high PV, and low wind-low PV scenarios, respectively).

**Table 1:** Predicted values for electricity load, heat load, hydrogen demand, wind power, and PV power.

Time (Hour)	Electricity load (MW)	Heat load (MWth)	Hydrogen demand (kg/h)	Wind power (MW)	PV power (MW)
1	150	120	50	80	0
2	140	110	45	85	0
3	130	100	40	90	0
4	125	95	38	95	0
5	130	100	40	100	0
6	140	110	45	95	5
7	160	130	55	90	20
8	180	150	60	85	50
9	200	170	70	80	80
10	220	190	75	75	100
11	240	210	80	70	120
12	250	220	85	65	130
13	245	215	82	70	125
14	230	200	78	75	110
15	210	180	72	80	90
16	190	160	65	85	60
17	180	150	60	90	30
18	170	140	58	95	10
19	160	130	55	100	0
20	170	140	58	95	0
21	180	150	60	90	0
22	170	140	58	85	0
23	160	130	55	80	0
24	150	120	50	75	0

The data in Table 1 represents a typical 24-hour profile for the proposed system, capturing the dynamic interactions between multi-energy demands and renewable generation sources under uncertainty constraints. Electricity load peaks at 250 MW during midday (hour 12), reflecting typical daytime industrial and commercial activity, while dropping to a minimum of 125 MW in the early morning (hour 4). Heat load follows a similar pattern, reaching 220 MWth at hour 12 and a low of 95 MWth at hour 4, indicating correlated thermal demands, possibly from heating or industrial processes. Hydrogen demand, ranging from 38 kg/h to 85 kg/h, aligns with load peaks, suggesting its role in energy storage or chemical production during high-demand periods to balance the system. Wind power exhibits an inverse correlation with loads, being stronger at night (peaking at 100 MW during hours 5 and 19) and weaker during the day (minimum 65 MW at hour 12), which highlights potential curtailment risks during off-peak hours without proper storage or coupling mechanisms. PV power, as expected, is solar-dependent, starting at 0 MW during non-sunlight hours, ramping up to a peak of 130 MW at hour 12, and tapering off in the evening. This complementarity between wind (night-dominant) and PV (day-dominant) supports multi-energy coupling, but uncertainties in their outputs (addressed via LHS scenarios) could lead to mismatches with loads, emphasizing the need for flexible units like BCCPP and P2A in the optimization model.

Overall, the profiles underscore the value of the proposed stochastic scheduling: high midday loads coincide with PV peaks but low wind, necessitating carbon capture and hydrogen production to absorb surpluses and reduce emissions. Simulation results show that adjusting carbon constraints can further lower emissions by 30–38%, demonstrating the model’s effectiveness in handling uncertainties and multi-energy synergies for low-carbon operation.

### 4.2 Low-carbon economic scheduling

Within the random low-carbon scheduling framework for MEVPP, the following four scenarios were designed to evaluate optimal low-carbon economic dispatch strategies. These scenarios progressively integrate low-carbon technologies, including biomass-coupled power generation, carbon capture, P2A, and electrochemical processes, in order to reduce system carbon emissions and improve economic efficiency.

Scenario 1: Baseline mode without carbon capture or other coupling technologies. The system relies only on conventional coal-fired units and wind power.
Scenario 2: Independent operation of coal-fired units with carbon capture. The energy consumption of the capture system is supplied only by thermal units, without wind power or P2A participation.
Scenario 3: Joint operation of wind power, carbon capture, and P2A. Part of the wind power is used for the capture system, and the captured CO₂ is further utilized for P2A to produce ammonia and urea via chemical synthesis.
Scenario 4: Based on Scenario 3, biomass is introduced to couple with coal-fired units. The system integrates the electricity–carbon–hydrogen–chemical process, achieving more efficient carbon recycling and producing hydrogen and chemical products.

Table 2 presents the cost comparison of the four scenarios, including fuel cost, wind curtailment penalty, carbon trading cost, total cost, and CO₂ emissions. All costs are expressed in US dollars ($) and represent a 24-hour operational period. The baseline system (Scenario 1) consists of conventional coal-fired units and wind power without carbon capture or multi-energy coupling technologies, operating under a carbon trading market with a price of 19.8 $/t CO₂. The total cost in Scenario 1 ($800,000) includes: fuel costs ($500,000) for coal consumption, wind curtailment penalties ($100,000) due to limited grid capacity, and carbon trading costs ($200,000) representing the cost of purchasing carbon emission allowances for 10,000 t of CO₂ emissions. The results indicate that progressive integration of low-carbon technologies significantly improves both economic and environmental performance. Compared with Scenario 1, the total cost in Scenario 2 decreases by 135,749.8 $ (17.0% reduction), with a 30.7% reduction in CO₂ emissions (from 10,000 t to 6,930 t). In Scenario 3, the total cost decreases further by 231,055.8$ (28.9% reduction from baseline), with a 33.0% reduction in emissions (to 6,697 t). Scenario 4 achieves the best results, reducing the total cost by $600,800 (75.1% reduction from baseline) and lowering CO₂ emissions by 38.5% (to 6,150 t). The cost reduction in Scenario 4 relative to Scenario 1 ($600,800) is primarily attributed to: (i) reduced fuel costs ($150,800 savings) due to biomass substitution and improved energy efficiency; (ii) elimination of wind curtailment penalties ($100,000 savings) through enhanced renewable integration; and (iii) carbon trading revenue shift from cost to income ($350,000 swing, from $200,000 cost in Scenario 1 to $150,000 revenue in Scenario 4, representing a total benefit of $350,000). These three components sum to the total cost reduction: $150,800 + $100,000 + $350,000 = $600,800. These improvements mainly result from the integration of carbon capture devices, which reduce CO₂ emissions from coal-fired units and enable revenue generation through carbon trading and CO₂ utilization. The sale of surplus carbon allowances generates revenue, turning carbon trading cost into a negative value. At the same time, the captured CO₂ is reused in P2A and electrochemical processes, enhancing the carbon–hydrogen–chemical coupling cycle.

**Table 2:** Comparison of costs and emissions under four scenarios.

Scenario	Fuel cost ($)	Wind curtailment penalty cost ($)	Carbon trading cost ($)	Total cost ($)	CO₂ emissions (t)
1	500,000	100,000	200,000	800,000	10,000
2	450,000	66,160	−50,000	466,160	6,930
3	400,000	0	−100,000	300,000	6,697
4	349,200	0	−150,000	199,200	6,150

The analysis reveals distinct mechanisms underlying performance improvements across scenarios. Compared to Scenario 1, Scenario 2 shows a wind curtailment penalty cost reduction of 33.84% (from $100,000 to $66,160). This improvement stems from two primary factors: (1) carbon capture systems require additional energy, reducing the net power output from thermal units and creating more grid capacity for wind integration; (2) the captured CO₂ can be stored and later utilized, creating operational flexibility. Scenario 3 demonstrates further improvements by introducing wind-carbon capture-P2A joint operation. The wind curtailment penalty cost drops to zero, and fuel costs decrease from $500,000 to $400,000, representing a 20% reduction. This occurs because surplus wind power drives the carbon capture system, and the captured CO₂ is utilized in P2A processes to produce ammonia and urea, generating additional revenue while consuming excess renewable energy. Scenario 4, which integrates biomass co-combustion and full electricity-carbon-hydrogen-chemical coupling, achieves the optimal performance. Fuel costs decrease by an additional 12.7% (from $400,000 to $349,200) compared to Scenario 3, primarily due to biomass substitution reducing coal consumption and the production of high-value chemical products. The fully coupled mode enhances renewable energy consumption, significantly reduces CO₂ emissions, and minimizes total system costs through synergistic energy utilization.

**Fig 5:**
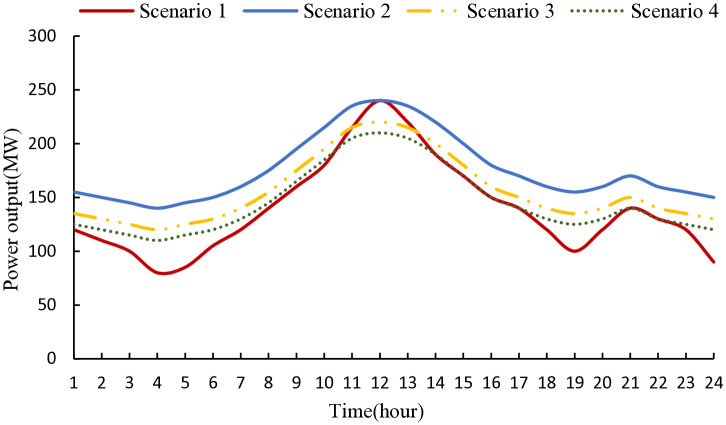
Output power of coal-fired units under four scenarios. The figure compares generation profiles under baseline operation, carbon capture integration, wind–carbon capture–P2A joint operation, and full electricity–carbon–hydrogen–chemical coupling with biomass co-combustion, illustrating the impact of low-carbon technologies on operational flexibility.

Fig 5 shows the output power of coal-fired units under four scenarios. After introducing the carbon capture system (Scenarios 2 and 3), the output of coal-fired units increases overall due to the need for extra energy for the carbon capture system, while the “peak-valley difference” in their output decreases. In Scenario 3, the output of coal-fired units decreases markedly during load valley periods because, in this joint operation mode, part of the excess wind power drives the carbon capture system, reducing the power demand from coal-fired units and thus lowering the system’s overall CO₂ emissions. In Scenario 4, the output of coal-fired units is further optimized due to the introduction of BCCPP and electricity-carbon-hydrogen-chemical multi-energy coupling, ensuring system flexibility while significantly improving carbon cycling efficiency and low-carbon operation levels.

### 4.3 Low-carbon economic operation under carbon emission constraints

To investigate the influence of carbon emission constraints on system operation, the optimized emission level obtained in Scenario 4 is regarded as the upper bound of CO₂ emissions. Emission reduction coefficients are set for optimization. The optimization results are summarized in Table 3.

**Table 3:** Costs and carbon capture amounts under different emission reduction coefficients.

Emission reduction coefficient	Total cost ($)	Carbon capture amount (t)
1.00	199,200	3,850
0.95	217,672	4,000
0.90	242,672	4,200
0.85	277,672	4,500
0.80	317,672	4,800
0.79	417,672	4,800
0.75	447,672	4,800

The results show that stricter carbon emission constraints increase CO₂ capture but also raise system costs. Total costs rise from 199,200 $ at coefficient 1.0 to 447,672 $ at 0.75, due to higher energy needs for capture and use of gas units. A sharp increase occurs between 0.8 (317,672 $) and 0.79 (417,672 $), where capture saturates at 4,800 t and the system shifts to costlier options for further reductions. This saturation behavior reflects the limitations of the BCCPP and multi-energy integration strategy, which enable efficient carbon cycling up to a certain point. Scenario 4 achieves the optimal trade-off, with maximum capture of 3,850 t at minimal incremental cost through biomass co-combustion. Additionally, Scenario 4 effectively reduces emissions through constraint adjustments, delivering the most balanced low-carbon operation under uncertainty. Table 4 shows the output power of coal-fired units, gas units, and carbon capture energy consumption under different carbon emission reduction coefficients.

**Table 4:** Unit output power and carbon capture energy consumption under different emission reduction coefficients (MW).

Emission reduction coefficient	Coal-fired unit output	Gas unit output	Carbon capture energy consumption
1.00	200	100	50
0.95	205	105	55
0.90	210	110	60
0.85	215	115	65
0.80	210	120	70
0.79	180	150	70
0.75	150	180	70

Table 4 illustrates the variations in unit output power and carbon capture energy consumption as the emission reduction coefficient decreases, reflecting the model’s response to stricter carbon constraints in a multi-energy coupled VPP. As the coefficient reduces, gas unit output and carbon capture energy consumption gradually increase, while coal-fired unit output initially rises slightly (up to coefficient 0.85) to provide additional energy for capture, then drops sharply below 0.85. This shift highlights the proposed system’s adaptation: coal-fired units, with higher CO₂ intensity, are curtailed in favor of lower-emission gas units to meet tighter limits.

### 4.4 Carbon trading price sensitivity analysis

**Fig 6:**
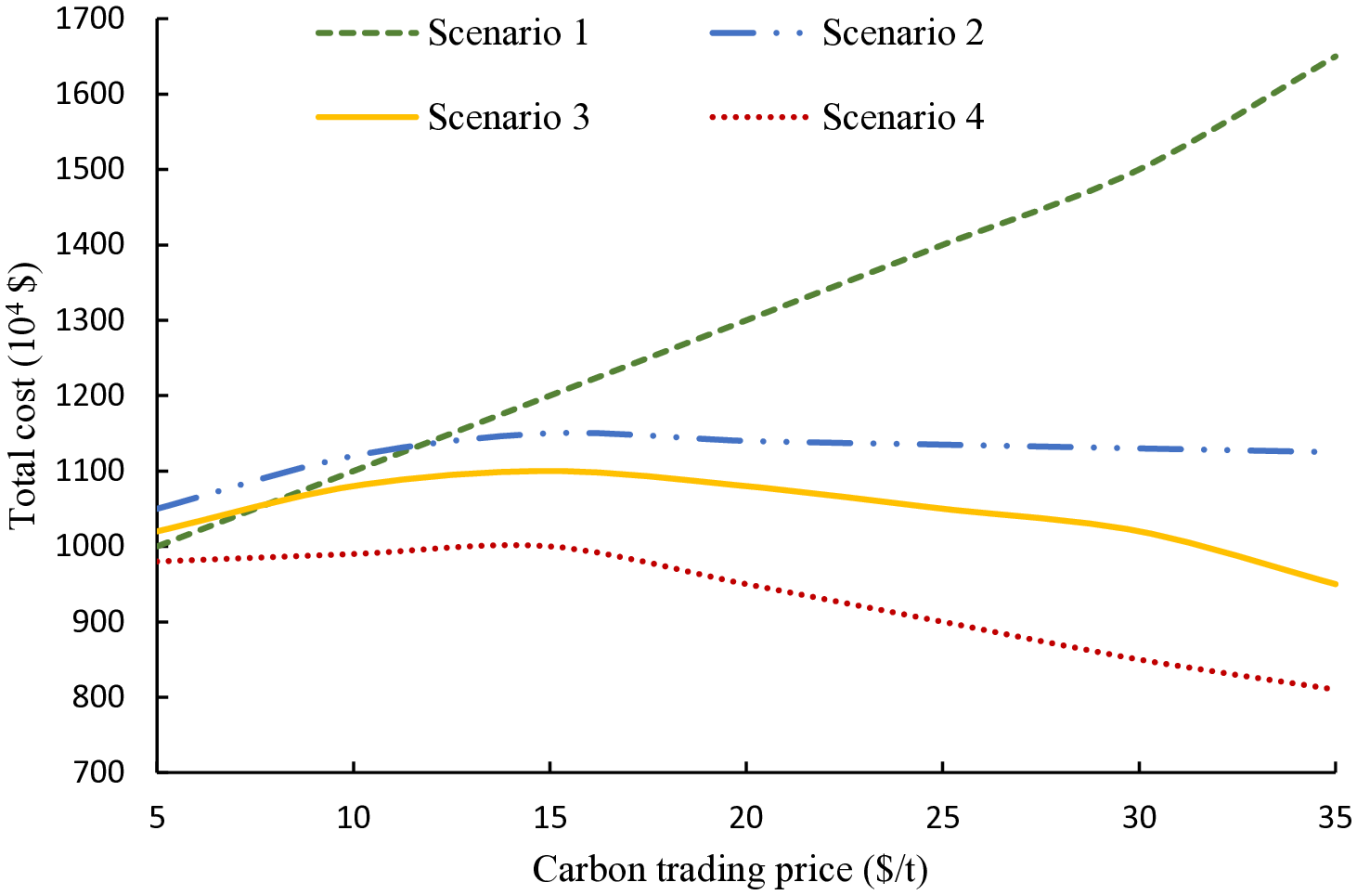
Total system costs under different carbon trading prices. The figure shows how system costs vary with increasing carbon prices across different operational scenarios, highlighting the economic impacts of carbon capture, renewable integration, and multi-energy coupling.

**Fig 7:**
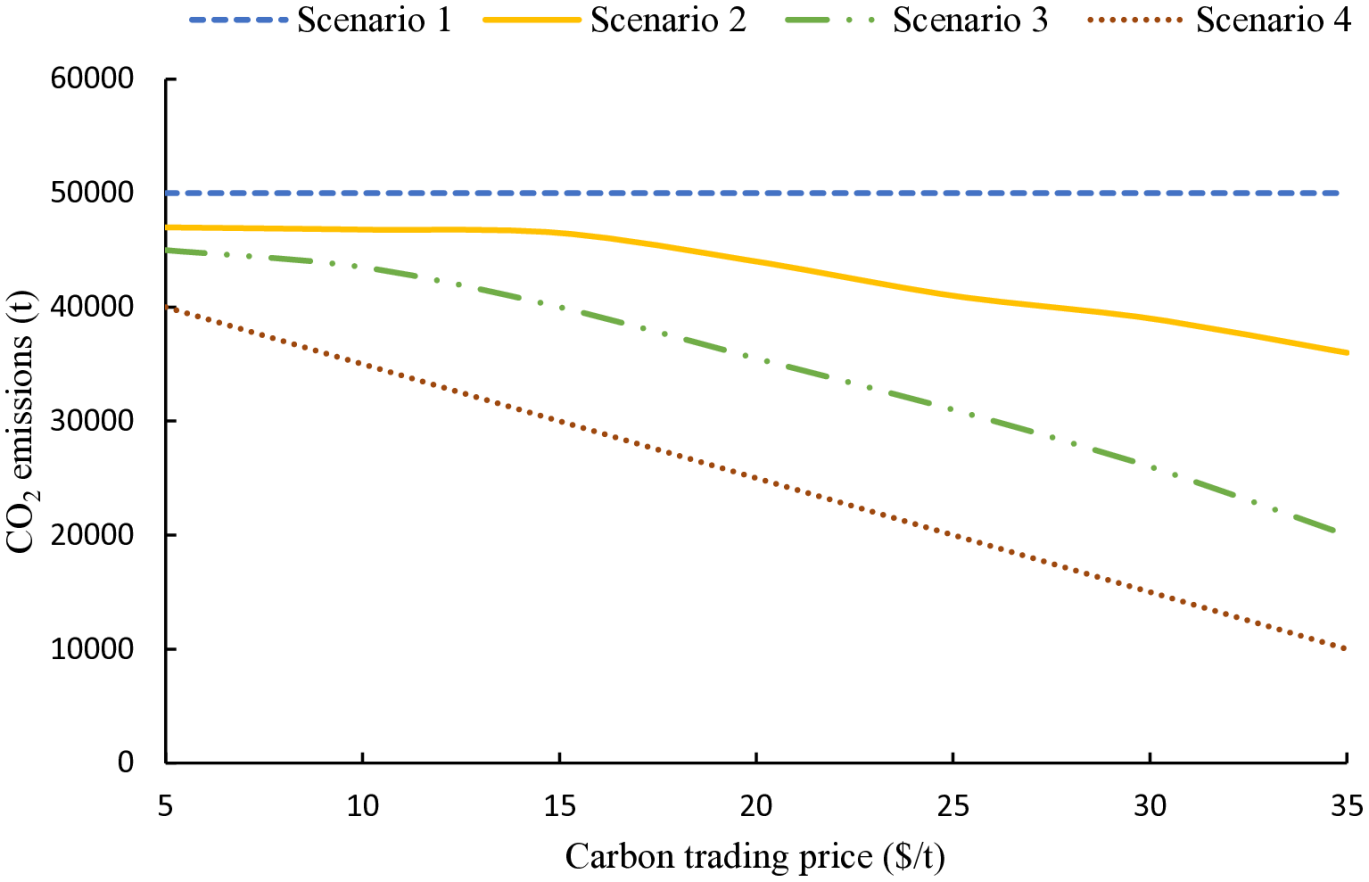
Carbon emission levels under different carbon trading prices. The figure compares emission responses of different scenarios as carbon prices increase, demonstrating the effectiveness of biomass co-combustion, carbon capture, and electricity–carbon–hydrogen–chemical coupling in reducing CO₂ emissions.

Under the carbon trading policy framework, variations in carbon trading prices exert a substantial influence on the low-carbon economic performance of the MEVPP system. This section simulates the effects on the system’s overall operation as carbon prices escalate from 5 $/t to 35 $/t. The resulting shifts in total system costs and carbon emissions are illustrated in Figs 6 and 7.

From Fig 6, it can be seen that in Scenario 1, lacking any capture mechanisms, costs escalate steadily with rising prices due to unmitigated emission penalties, showing no adaptive response. Scenario 2 incorporates carbon capture powered solely by thermal units, where costs increase at low prices (below 10 $/t) but stabilize around 20 $/t as capture becomes viable, leading to modest cost offsets from reduced trading fees. Scenario 3 enhances this by leveraging wind power for capture energy, resulting in earlier cost moderation (starting at 8 $/t) and more substantial declines at higher prices, as P2A utilization of captured CO₂ generates additional revenue from ammonia and urea via chemical synthesis production. Scenario 4 delivers superior outcomes, with biomass co-combustion enabling efficient carbon recycling; costs are least affected at low prices and decrease sharply beyond 15 $/t through integrated hydrogen and chemical outputs, achieving the lowest overall costs by maximizing multi-energy synergies and minimizing waste.

Fig 7 presents the corresponding carbon emission trends. Scenario 1 maintains consistently high emissions across the price range, as no mitigation technologies are employed. In Scenario 2, emissions hold steady until prices surpass 15 $/t, after which capture activation yields a gradual reduction. Scenario 3 shows improved results, with emissions declining earlier (from 10 $/t) and more steeply, thanks to wind-powered capture and P2A chemical synthesis that repurposes CO₂, effectively lowering the system’s carbon footprint while addressing wind curtailment. Scenario 4 exhibits the best performance, featuring significant emission drops even at 5 $/t due to biomass integration and full electricity-carbon-hydrogen-chemical coupling; at elevated prices, emissions plummet to minimal levels, demonstrating enhanced recycling efficiency and uncertainty management via stochastic optimization.

### 4.5 Comparison with alternative optimization approaches

To evaluate the efficacy of the proposed model, a comparative analysis is conducted against alternative optimization approaches commonly used in VPP scheduling. The deterministic optimization approach, which uses point forecasts without uncertainty consideration, serves as a baseline comparison. Additionally, a robust optimization method with uncertainty sets is evaluated.

The key performance metrics, including total cost in Scenario 4, cost increase relative to the proposed stochastic model, and computational time (on the specified hardware for a 24-hour horizon with 4 scenarios), are summarized in Table 5.

**Table 5:** Comparison of Optimization Approaches in Scenario 4.

Approach	Total cost ($)	Cost increase (%)	Computational time (s)
Proposed Stochastic (LHS-based)	199,200	–	45
Deterministic	320,000	60.7	20
Robust	280,000	40.5	60

It can be observed from Table 5 that the deterministic approach achieves a total cost of $320,000 in Scenario 4, which is 60.7% higher than the proposed stochastic model ($199,200), primarily due to its inability to hedge against renewable uncertainties, leading to higher wind curtailment penalties and suboptimal multi-energy coordination. The robust optimization approach, while providing worst-case guarantees, results in a total cost of $280,000, which is 40.5% higher than the proposed model, reflecting the conservatism inherent in robust formulations that prioritize risk mitigation over expected performance. The proposed LHS-based stochastic optimization approach balances computational efficiency with solution quality, achieving superior economic performance while maintaining reasonable computational times (approximately 45 seconds for a 24-hour horizon with 4 scenarios on the specified hardware).

## 5 Conclusion

This study develops and validates an optimization scheduling model for MEVPP that integrates uncertainty constraints with multi-energy coupling. The key novelty lies in combining BCCPP with P2A and low-carbon chemical production (e.g., urea synthesis) in a stochastic framework, extending prior models [31–33] by enabling carbon recycling and achieving 38.5% emission reductions (from 10,000 t to 6,150 t) and 75.1% cost savings (from $800,000 to $199,200) over baselines in a 24-hour period. The simulation results validate the efficacy of the proposed MEVPPs in balancing economic performance and low-carbon objectives under uncertainty. By integrating BCCPPs, CHP units, renewables, P2A processes, and chemical production, the model achieves significant reductions in carbon emissions, while enhancing renewable energy accommodation and system flexibility through multi-energy synergies.

Sensitivity analyses show that carbon prices above 15 $/t incentivize capture and recycling, stabilizing costs and minimizing emissions, with Scenario 4 outperforming others across 5–35 $/t. These results align with objectives of low-carbon economic scheduling and multi-energy coordination, offering superior renewable integration compared to electricity-focused models. Broader implications include guiding system operators in transitioning coal plants to multi-energy hubs for grid stability and zero curtailment, while policymakers can use tiered carbon pricing (>15 $/t) to drive reductions, supporting China’s dual-carbon goals and transferable to coal-reliant nations. This framework not only mitigates uncertainties associated with wind and photovoltaic outputs through Latin hypercube sampling–based stochastic optimization but also promotes efficient carbon recycling, offering practical insights for policymakers and operators in advancing China’s dual-carbon goals. The approach is transferable to other countries with similar energy transition challenges, particularly those with significant coal capacity and renewable energy potential.

Several limitations of this study should be noted. First, the analysis relies on simulated data without real-world validation; future work should evaluate the proposed framework using empirical data from pilot projects to validate model accuracy and transferability. Second, the model adopts an expected value–based optimization approach and does not explicitly assess scenario-level variance or risk; incorporating risk-aware metrics (e.g., Conditional Value at Risk) or robust optimization is a promising direction for future research. Third, chemical conversion processes are represented using efficiency-based, steady-state models for computational tractability, which do not capture detailed dynamic behaviors or transient responses; future work could incorporate dynamic process models to enhance realism. Fourth, the current framework focuses on day-ahead scheduling with simplified uncertainty and market representations; future extensions may include multi-timescale optimization (day-ahead, intraday, real-time), enhanced market participation strategies, and additional flexibility resources such as demand response and advanced energy storage technologies. Additionally, practical implementation challenges include computational scalability, as the MILP with LHS scenarios may become intractable for larger systems, requiring advanced solvers or decomposition; data requirements, limited by privacy and inaccuracies in uncertainty distributions and real-time parameters; and integration barriers, involving high costs and regulatory hurdles for retrofitting coal units to BCCPP and ensuring unit interoperability. These highlight the need for pilot demonstrations to bridge theory and practice.
